# Subunit and frequency-dependent inhibition of Acid Sensing Ion Channels by local anesthetic tetracaine

**DOI:** 10.1186/1744-8069-9-27

**Published:** 2013-06-10

**Authors:** Tiandong Leng, Jun Lin, James E Cottrell, Zhi-Gang Xiong

**Affiliations:** 1 Neuroscience Institute, Morehouse School of Medicine, Atlanta, GA 30310, USA; 2 Department of Anesthesiology, SUNY Downstate Medical Center, Brooklyn, NY 11203, USA

**Keywords:** ASIC, Local anesthetics, Tetracaine, Frequency-dependent inhibition

## Abstract

**Background:**

Extracellular acidosis is a prominent feature of multiple pathological conditions, correlating with pain sensation. Acid-sensing ion channels (ASICs), a family of proton-gated cation channels, are distributed throughout the central and peripheral nervous systems. Activation of ASICs, particularly ASIC3 and ASIC1a channels, by acidic pH and the resultant depolarization of nociceptive primary sensory neurons, participates in nociception. Agents that inhibit the activation of ASICs are thus expected to be analgesic. Here, we studied the effect of local anesthetic tetracaine on ASIC currents.

**Results:**

Tetracaine inhibited the peak ASIC3 current in a concentration-dependent manner with an IC_50_ of 9.96 ± 1.88 mM. The degree of inhibition by tetracaine was dependent on the extracellular pH but independent of the membrane potential. Furthermore, 3 mM tetracaine also inhibited 29.83% of the sustained ASIC3 current. In addition to ASIC3, tetracaine inhibited the ASIC1a and ASIC1β currents. The inhibition of the ASIC1a current was influenced by the frequency of channel activation. In contrast to ASIC3, ASIC1a, and ASIC1β currents, ASIC2a current was not inhibited by tetracaine. In cultured mouse dorsal root ganglion neurons, 1–3 mM tetracaine inhibited both the transient and sustained ASIC currents. At pH4.5, 3 mM tetracaine reduced the peak ASIC current to 60.06 ± 4.51%, and the sustained current to 48.24 ± 7.02% of the control values in dorsal root ganglion neurons. In contrast to ASICs, voltage-gated sodium channels were inhibited by acid, with 55.15% inhibition at pH6.0 and complete inhibition at pH5.0.

**Conclusions:**

These findings disclose a potential new mechanism underlying the analgesic effects of local anesthetics, particularly in acidic conditions where their primary target (i.e. voltage-gated Na^+^ channel) has been suppressed by protons.

## Background

Acid-Sensing Ion Channels (ASICs), members of the degenerin/epithelial sodium channel (Deg/ENaC) superfamily, are abundantly distributed in the central and peripheral neurons [1,2]. All ASICs contain two hydrophobic transmembrane domains surrounding a large extracellular loop and relatively short intracellular NH_2_- and COOH- terminal domains [3]. In rodents, there exist at least six ASIC subunits including ASIC1a, ASIC1b(β), ASIC2a, ASIC2b, ASIC3 and ASIC4 [4]. ASIC1a and ASIC2a are abundant in the central and peripheral nervous system while ASIC3 and ASIC1b are restricted to the peripheral nervous system [5-7]. ASICs are H^+^-gated channels sensitive to acidic pH to a varying extent depending on the subunit composition of the channels. For example, ASIC1a and ASIC3 are very sensitive to protons with an activation threshold close to pH7.0. ASIC1a has a pH_0.5_ of ~6.2 and mediates fast decaying, transient currents [8]. ASIC3 has two current components including a peak component with a pH_0.5_ of ~6.2 and a sustained current component with a pH_0.5_ of ~4.3 [5,9]. Similar to ASIC1a, ASIC1b has a pH_0.5_ of ~6.0 and mediates a transient current [6,10]. ASIC2a has low sensitivity to acidic pH with a pH_0.5_ of ~4.4 [11]. ASIC2b and ASIC4 do not show functional channel activity on their own [12-14]. Activation of ASICs by protons induces sodium (and calcium for homomeric ASIC1a channels) influx, resulting in membrane depolarization and neuronal excitation. Several studies have shown that ASICs play important roles in physiological processes such as nociception [15-19], synaptic plasticity and learning/memory [20], and in pathological conditions such as brain ischemia [21-24], seizure [25], multiple sclerosis [26], and tumor cell migration [27,28].

In painful conditions such as ischemia, skin and muscle incision, arthritis and inflammation, protons are produced or released by the injured tissues, resulting in tissue acidosis [4,29,30]. For example, the fall of local pH to 5.4 in inflammation and to 4.7 in fracture-related hematomas have been documented [31]. It has been demonstrated that accumulations of protons depolarize the terminals of nociceptive primary sensory neurons to cause pain sensation, and that the depolarization is caused by a direct activation of proton gated ionic channels [32,33]. Although both ASICs and Transient Receptor Potential Vanilloid receptor type 1 (TRPV1) could be involved, recent studies have suggested that ASICs, rather than TRPV1, mediate pain sensation induced by acid injection [17,34]. Although ASIC1a and ASIC3 have been implicated in acute pain sensation, ASIC3, the subunit that conducts both transient and sustained currents [5], may have a unique role in pain sensation in chronic conditions [17,29,35].

Local anesthetics have multiple effects including antinociception and analgesia, antiarrhythmia, and neuroprotection [36-38]. Blockage of voltage-gated sodium channels is a well-known and medically important mechanism of local anesthetics [39]. However, other mechanisms are likely to be involved particularly in the conditions of severe acidosis where the activities of voltage-gated sodium channels are already diminished by acidic pH [40-42]. Since ASICs in peripheral sensory neurons are implicated in nociception, and our previous studies showed an inhibitory effect of lidocaine on ASICs in mouse cortical neurons [43], we hypothesize that local anesthetics such as tetracaine might suppress the ASIC currents mediated by ASIC subunits that are highly and/or preferentially expressed in peripheral primary sensory (e.g. DRG) neurons. Tetracaine was approved and is still used as local anesthetic, which has the potential in neuraxial anesthesia or infiltrative anesthesia. Compared to lidocaine as well as other local anesthetics, tetracaine has a longer duration, particularly in the presence of a constrictor, which may render tetracaine a desirable local anesthetic alone or combined with other local anesthetics, in plexus/major nerve block for acute or chronic pain management. Here, we demonstrate that tetracaine inhibits ASIC currents expressed in Chinese hamster ovary (CHO) cells and in native dorsal root ganglion (DRG) neurons in a concentration range that can be reached for nerve blockade [44]. This finding discloses a potential new mechanism underlying the analgesic effects of tetracaine.

## Results

### Tetracaine inhibits ASIC3 current in a reversible and concentration-dependent manner

**Figure 1:**
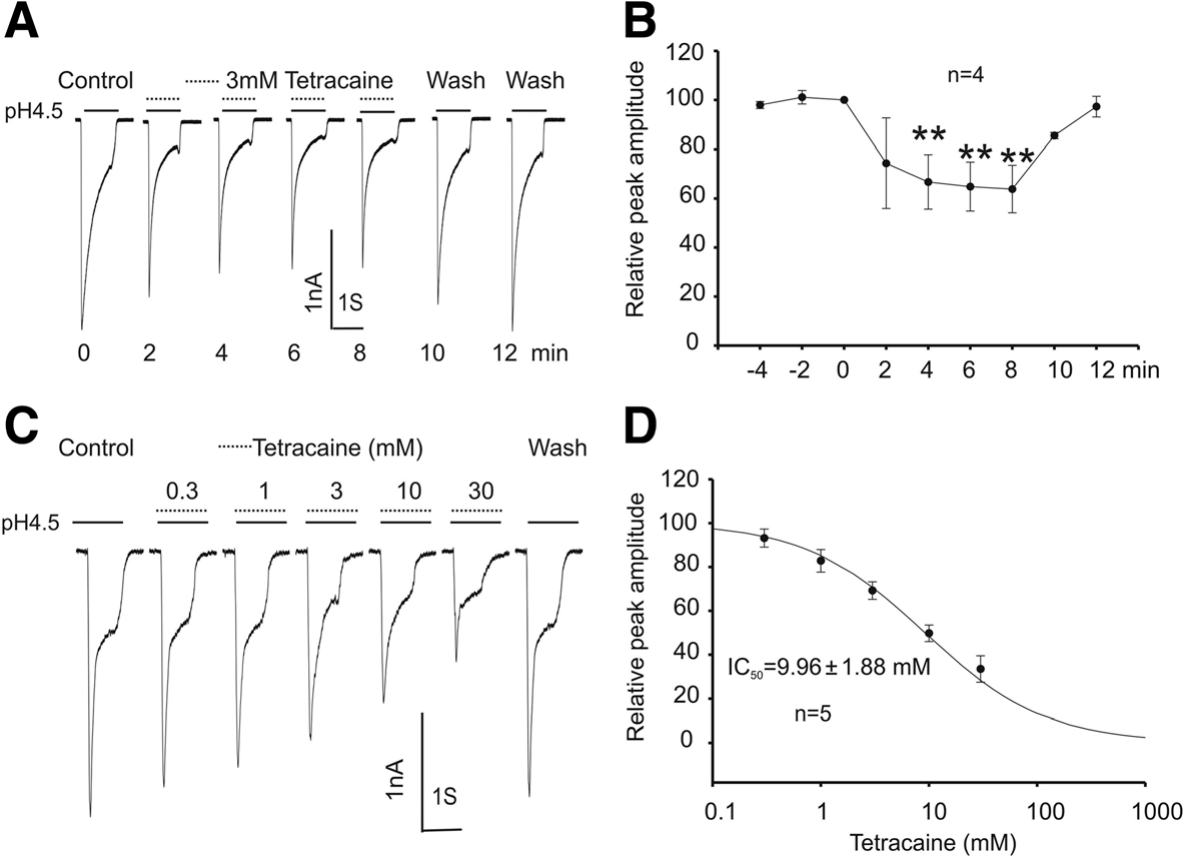
**Reversible and concentration**-**dependent inhibition of ASIC3 currents by tetracaine. A**, **B**. Representative current traces and summary data showing reversible inhibition of the ASIC3 current expressed in Chinese Hamster Ovary cells (CHO) cells. ASIC3 current was induced by decreasing the extracellular pH from 7.4 to 4.5. Tetracaine was added to the pH 4.5 solution at a final concentration of 3 mM (Paired *t* test, *n* = 4; **p < 0.01). **C**, **D**. Representative current traces and summary data showing a concentration-dependent inhibition of the peak amplitude of ASIC3 currents by tetracaine. The IC_50_ value was 9.96 ± 1.88 mM (*n* = 5). Data were expressed as mean ± SD.

In cultured CHO cells transfected with ASIC3 subunit, a rapid reduction of pH_o_ from 7.4 to 4.5 for 1 second evoked a large transient inward current (Figure 1A). Following the recording of stable ASIC3 currents, the effect of tetracaine was tested by adding different concentrations of tetracaine to the acidic solution. Addition of 3 mM tetracaine rapidly inhibited the peak current by 35.18% (n = 4, p < 0.01), and the inhibitory effect was reversible after a short period of washout (Figure 1A-B). At a lower concentration of 1 mM, a concentration expected to be reached in clinical dosage range [45,46], the amplitude of the ASIC3 current was reduced by 17.17% (p < 0.05, n = 4). More detailed dose–response analysis yielded a threshold concentration of 0.3 mM (6.82% inhibition) and a half-maximal inhibitory concentration of 9.96 ± 1.88 mM (n = 5, Figure 1C-D). For unknown reason, higher concentration of tetracaine (100 mM) caused an immediate loss of the tight seal in all cells recorded (n > 5, data not shown).

### Tetracaine inhibits ASIC3 current in a pH-dependent and voltage-independent manner

**Figure 2:**
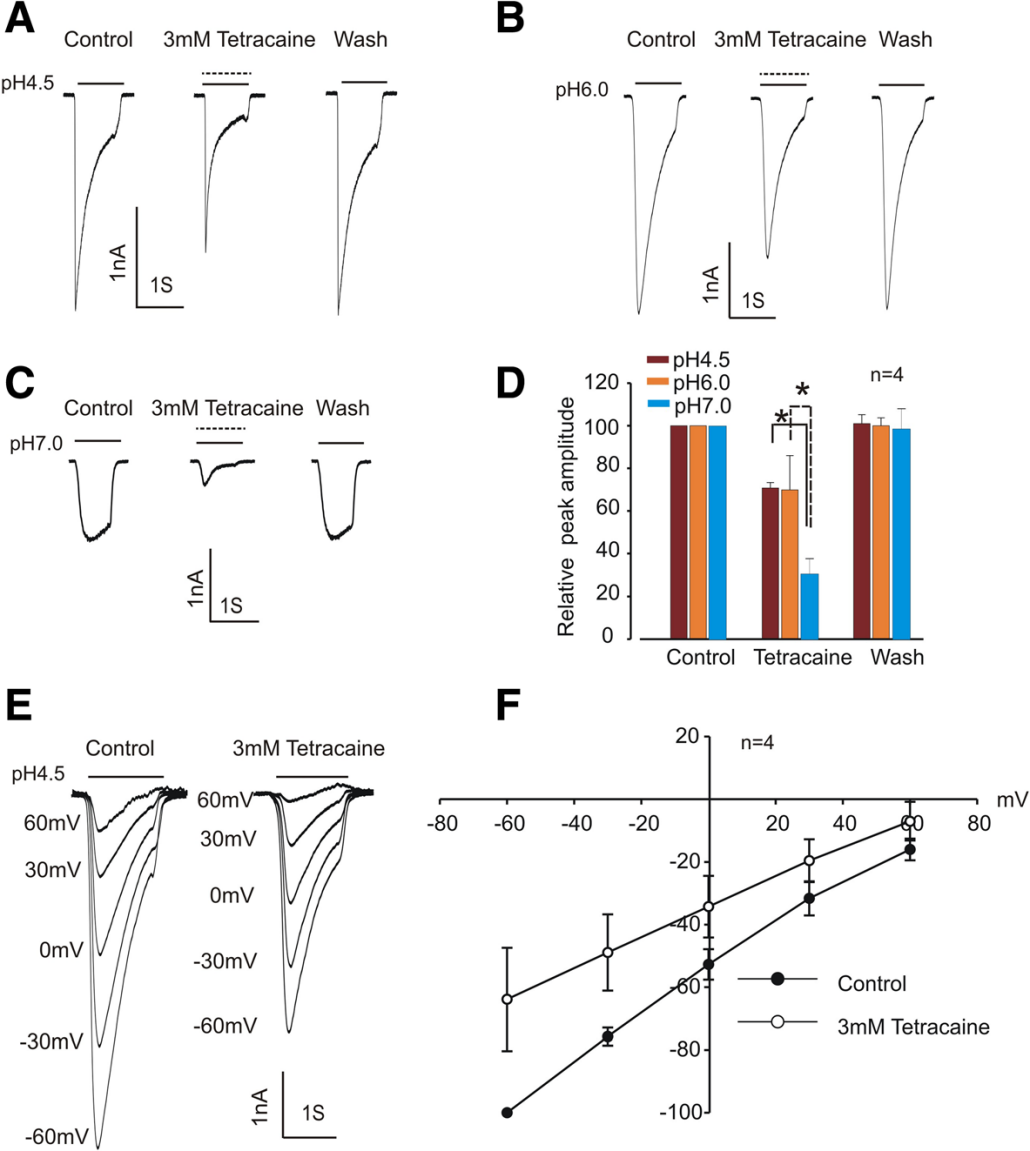
**pH**-**dependent and voltage**-**independent inhibition of ASIC3 currents by tetracaine. A**, **B**, **C**. Representative current traces showing inhibition of ASIC3 currents by 3 mM tetracaine at pH4.5, pH6.0, and pH7.0, respectively. **D**. Summary data showing inhibition of the ASIC3 current by 3 mM tetracaine at different pH levels as indicated (*t* test, *n* = 4; *p < 0.05). **E**. Representative current traces showing ASIC3 current activated at different holding potentials ranging from −60 to +60 mV in the absence and presence of 3 mM tetracaine. **F**. Current–voltage relationship (*I*-*V* curve) in the absence and presence of 3 mM tetracaine; *n* = 4.

Most local anesthetics are tertiary amines with pKa in the range of 7 to 10. The fractions of uncharged free amines and cationic protonated forms change with pH [47]. Under acidic conditions, more protonated forms of local anesthetics are available, which might limit their inhibitory potency, as shown for voltage-gated sodium channels. To determine whether the effect of tetracaine on the ASIC3 current is also pH-dependent, we compared the inhibitory effect of tetracaine on ASIC currents activated at pH4.5, pH6.0 and pH7.0. We found no significant difference in the effect of tetracaine on ASIC currents between pH6.0 and pH4.5 (30.16% inhibition at pH6.0 and 29.43% inhibition at pH4.5, n = 4) (Figure 2A-B, D). However, a 69.30% inhibition was observed at pH7.0, which is significantly greater than those at pH6.0 and pH4.5 (Figure 2C-D). These results suggest a pH-dependent inhibition of the ASIC current by tetracaine. To determine whether the inhibition of ASIC3 currents by tetracaine is voltage-dependent, currents evoked by lowering pH_o_ from 7.4 to 4.5 were recorded over a range of holding potentials from −60 to +60 mV with an increment of 30 mV (Figure 2E). As shown in Figure 2F, the current–voltage relationships were linear in the absence and presence of 3 mM tetracaine (n = 4), demonstrating a lack of voltage dependence.

### Tetracaine inhibits the sustained ASIC3 current

**Figure 3:**
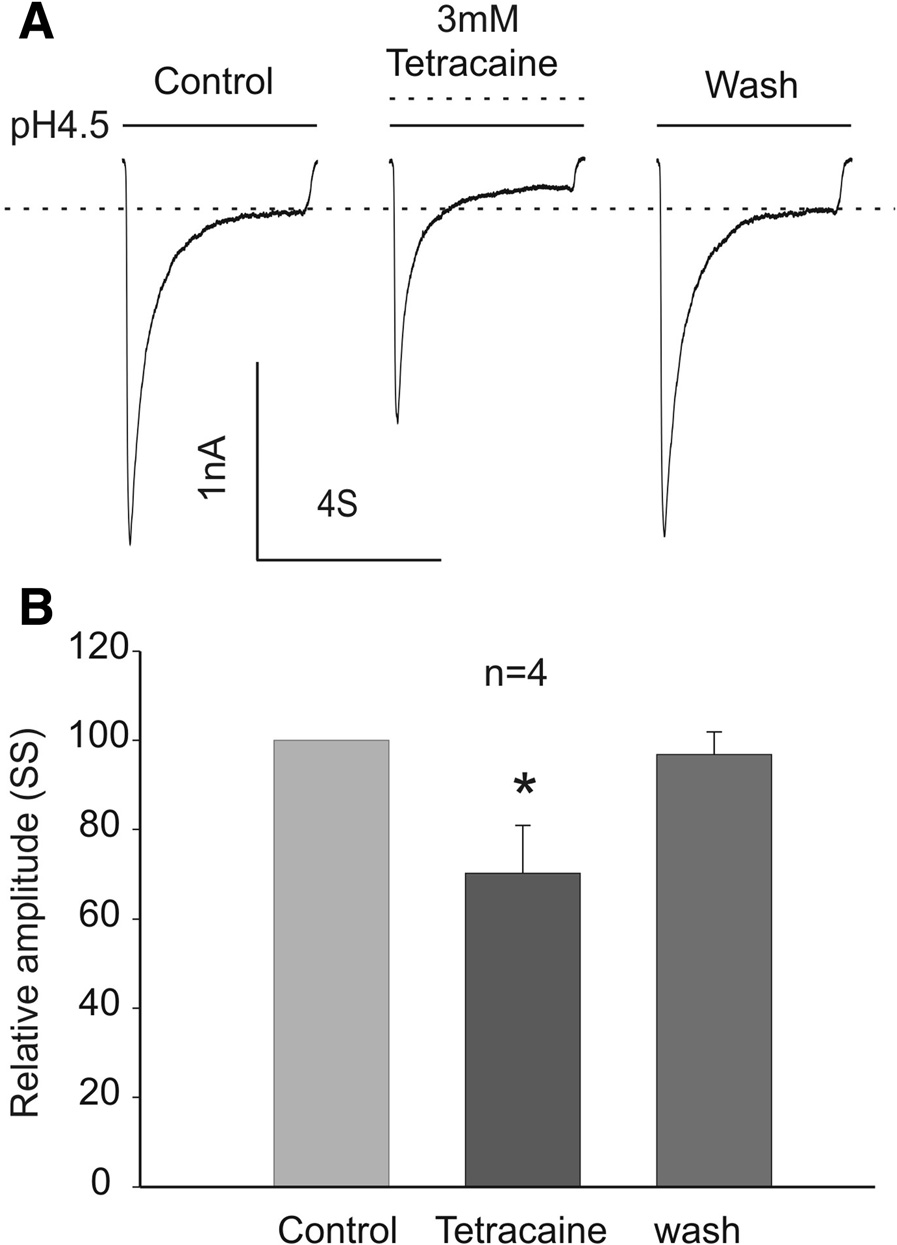
**Inhibition of sustained ASIC3 current by tetracaine. A**, **B**. Representative current traces and summary data showing a reversible inhibition of the steady state (SS) component of ASIC3 current activated at pH 4.5 by 3 mM tetracaine (paired *t* test, *n* = 4; *p < 0.05).

ASIC3 current is characterized by a fast desensitizing early component followed by a sustained non-desensitizing late component [4]. The sustained component, which persists in the continuous presence of tissue acidosis, likely contributes to prolonged depolarization of peripheral sensory neurons and sensation of nonadapting pain [48,49]. To investigate the potential inhibitory effect of tetracaine on the non-desensitizing component of the ASIC3 current, we perfused the cells with a pH 4.5 solution for 4 seconds to evoke the sustained ASIC3 current (Figure 3A). As shown in Figure 3A-B, 3 mM tetracaine significantly and reversibly decreased the amplitude of the sustained current by ~30% (n = 4, Figure 3A-B).

### Tetracaine inhibits the ASIC1a current in a frequency-dependent manner

**Figure 4:**
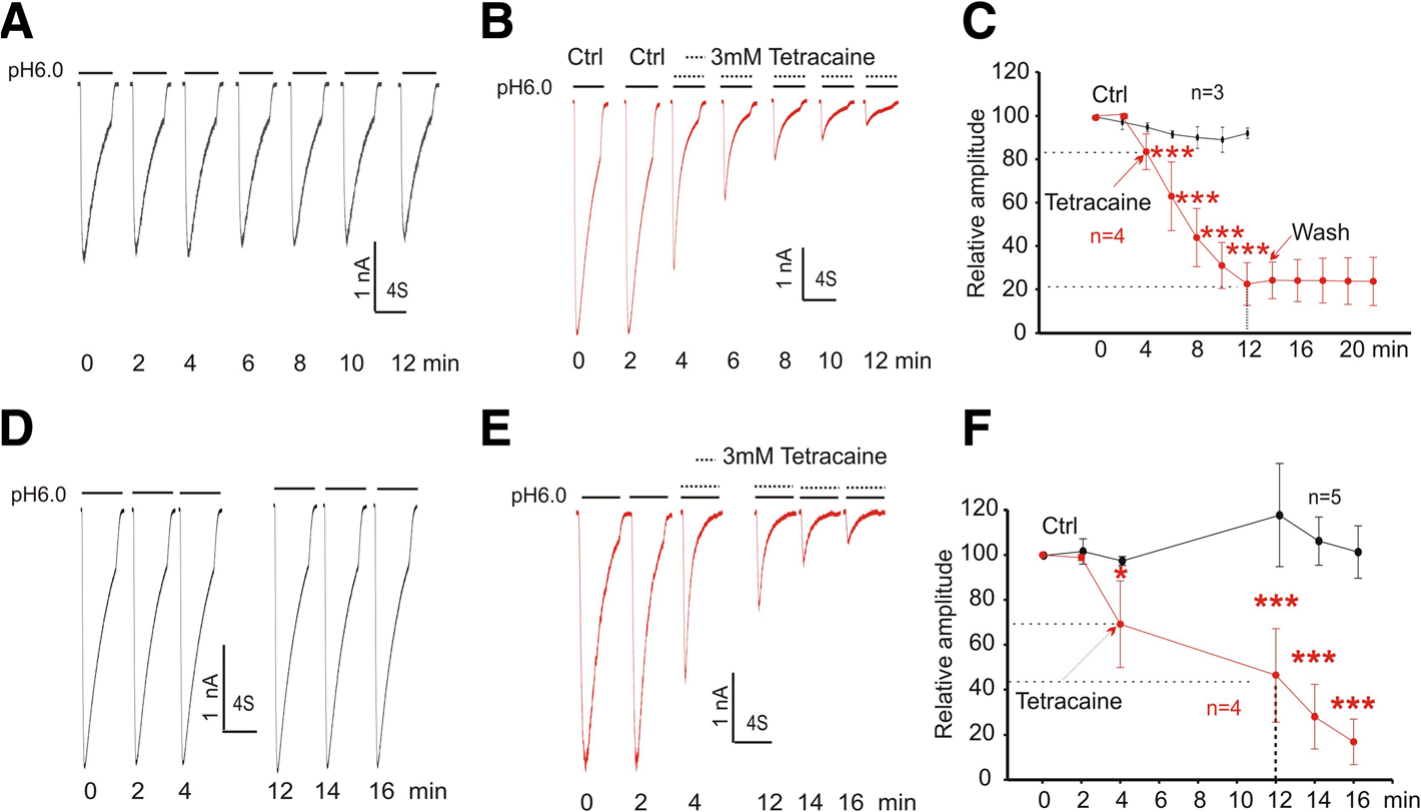
**Inhibition of the ASIC1a current by tetracaine. A**, **B** and **C**. Representative current traces and summary data showing time-dependent decrease of ASIC1a current after rundown in the absence (*n* = 3, black) and presence (*n* = 4, red) of 3 mM tetracaine (Two-way ANOVA followed by Bonferroni posttests, ***p < 0.001). **D**, **E** and **F**. Representative current traces and summary data showing the frequency-dependent inhibition of the ASIC1a current in the absence (*n* = 5, black) and presence (*n* = 4, red) of 3 mM Tetracaine; (Two-way ANOVA followed by Bonferroni posttests, *p < 0.05; ***p < 0.001).

ASIC1a is one of the most sensitive pH sensors in the central and peripheral nervous system. Similar to ASIC3 channels, activation of ASIC1a channels has been implicated in pain sensation [8,16,18,19]. Therefore, we also determined whether tetracaine has an inhibitory effect on the ASIC1a current. When pH_o_ was decreased from 7.4 to 6.0, a transient and prominent inward current was induced in CHO cells transfected with complimentary DNA encoding ASIC1a subunit (Figure 4A-B). In most cells, the amplitude of ASIC1a currents decreased gradually within the first 10–15 min after formation of whole-cell configuration but stabilized within ~20 min. Unless otherwise stated, we only tested the effect of tetracaine 20 min after the formation of whole-cell recording and following the recording of at least three stable currents. With 3 mM tetracaine added in the acidic solution, the ASIC1a current decreased progressively with repeated activation of the channels (Figure 4B). 10 min after the start of tetracaine exposure, approximately 80% of the ASIC1a current was inhibited (Figure 4C). The current did not recover after ~10 min washout (Figure 4C). In the absence of tetracaine, as a control, the ASIC1a current was relatively stable within the same time period (Figure 4A, C).

Since tetracaine was only present in the low pH solution and applied to cells for a few seconds every 2 min, the gradual and time-dependent inhibition of the ASIC1a current by tetracaine suggested a possibility of frequency-dependency.

To provide more evidence of frequency-dependent inhibition, we reduced frequency of channel activation from 5 times (Figure 4A-B) to 2 times (Figure 4D-E) during the same time period from 4 min to 12 min. We found that the inhibitory effect of tetracaine was significantly reduced (Figure 4E-F). In the same paradigm, there was no reduction of the ASIC1a current in the absence of tetracaine (Figure 4D). Thus, the inhibition of the ASIC1a current by tetracaine is frequency-dependent. The higher frequency the channel opens, the more inhibition takes place.

### Effect of tetracaine on ASIC1β and ASIC2a currents

**Figure 5:**
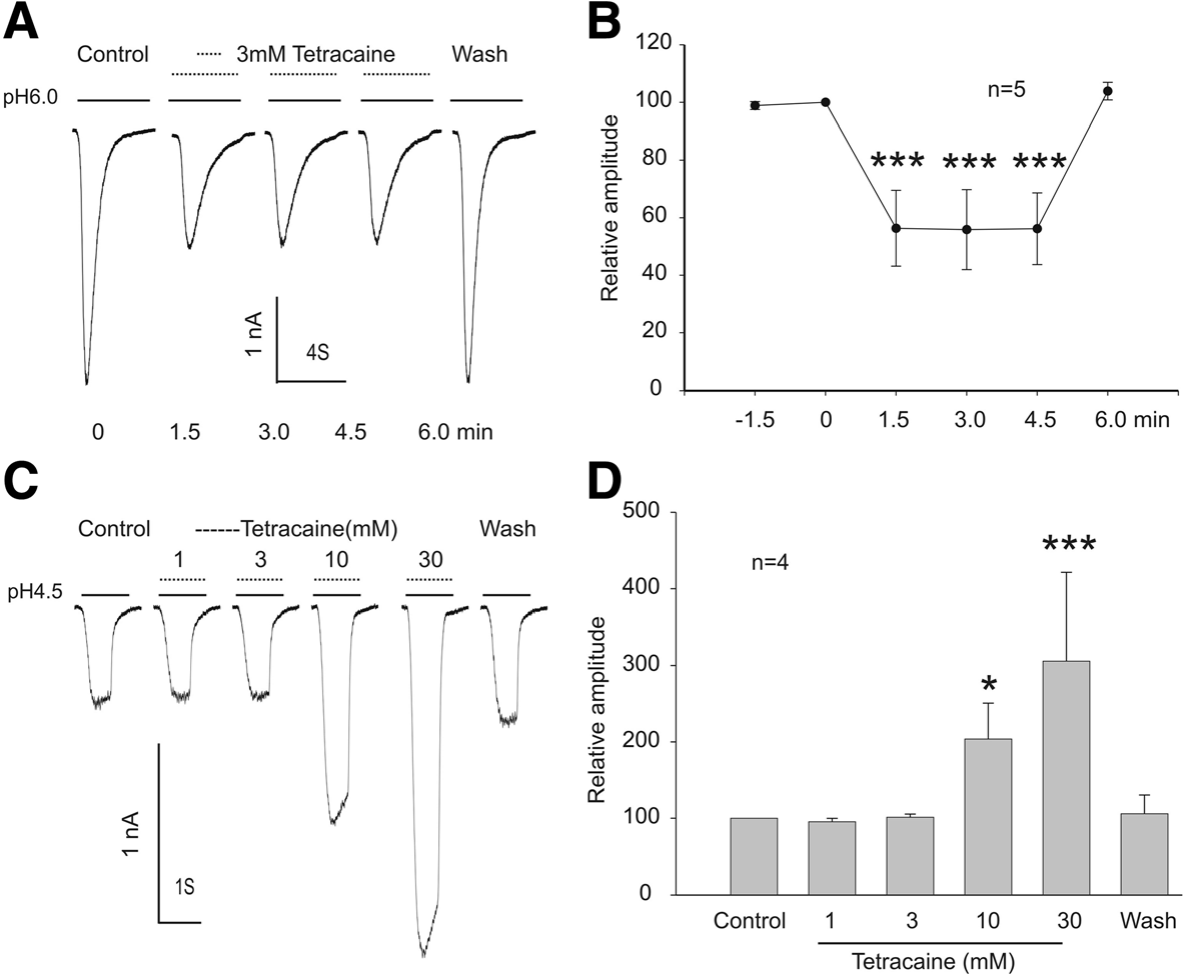
**Effect of tetracaine on ASIC1β and ASIC2a current. A**, **B**. Representative current traces and summary data showing the inhibition of ASIC1β current expressed in Chinese Hamster Ovary cells (CHO) cells by 3 mM tetracaine; (Paired *t* test, *n* = 5; ***p < 0.001). **C**, **D**. Representative current traces and summary data showing the potentiation of the ASIC2a current in Chinese Hamster Ovary cells (CHO) cells by 10 and 30 mM tetracaine (paired *t* test, *n* = 4; *p < 0.05, ***p < 0.001).

To further determine whether the effect of tetracaine is subunit-dependent, we also investigated the effect of tetracaine on ASIC1β and ASIC2a currents. As shown in Figure 5A, lowering pH_o_ to 6.0 induced a transient inward ASIC1β current. Addition of 3 mM tetracaine in the acidic solution rapidly and reversibly decreased the current amplitude by 52% (Figure 5B, n = 5). Unlike ASIC1a, the inhibition of the ASIC1β current by tetracaine does not show clear time or frequency-dependency. In contrast to ASIC3, ASIC1a, and ASIC1β, ASIC2a current was not affected by 1–3 mM tetracaine (Figure 5C). Interestingly, higher concentrations (>10 mM) of tetracaine potentiated the ASIC2a current (Figure 5C-D). We were, however, unable to test the effect of 100 mM tetracaine on any subunit of ASICs since it caused an immediate loss of tight seal for an unknown reason.

### Effect of tetracaine on acid-evoked current in primary cultured DRG neurons

**Figure 6:**
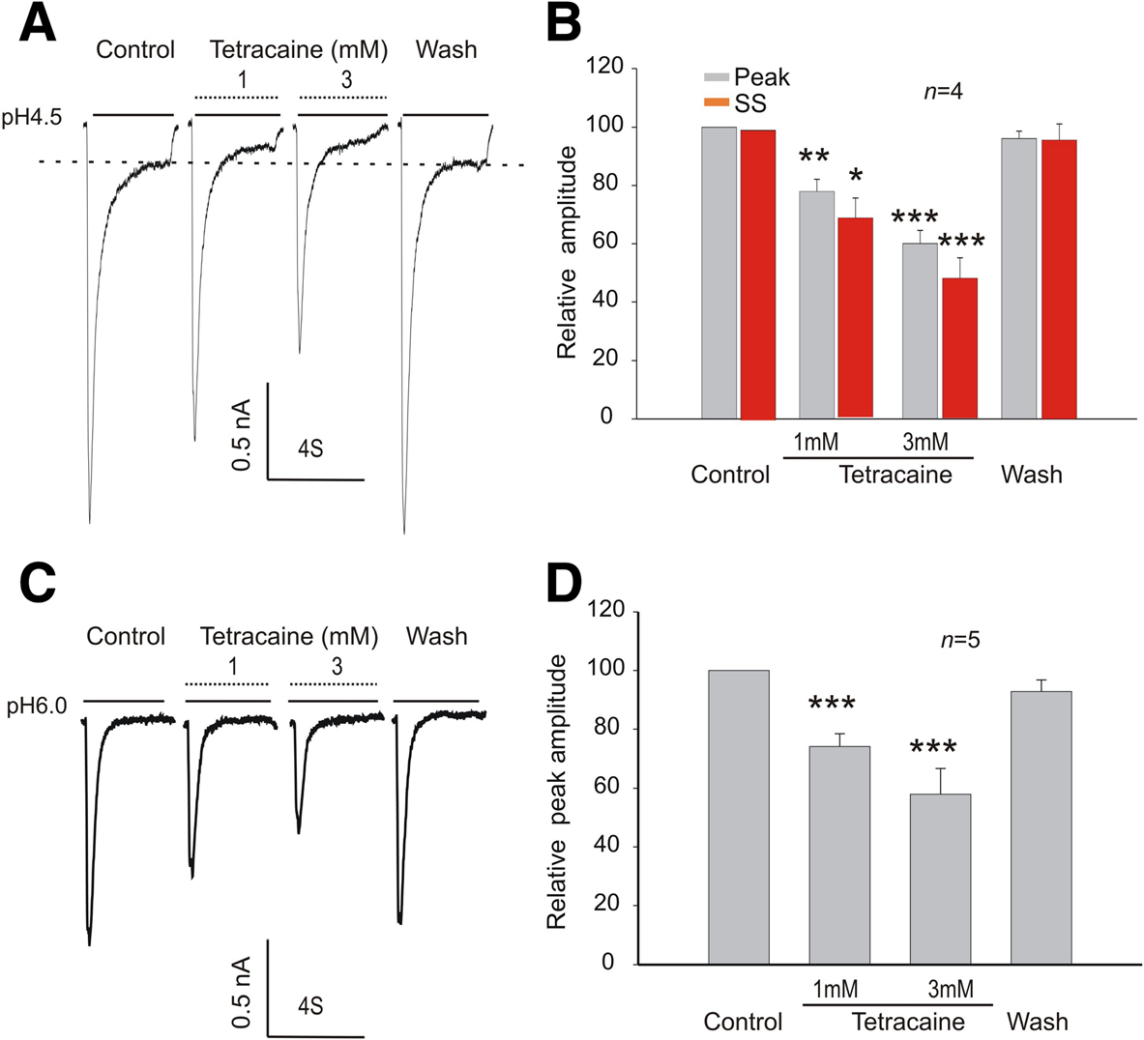
**Inhibition of ASIC3-like current in dorsal root ganglion (DRG) neurons by tetracaine. A**, **B**. Representative current traces and summary data showing a reversible inhibition of the peak and steady state (SS) components of the ASIC3-like current activated at pH 4.5 by 1 mM and 3 mM tetracaine (paired *t* test, *n* = 4; *p < 0.05, **p < 0.01, ***p < 0.001). **C**, **D**. Representative current traces and summary data showing a reversible inhibition of the amplitude of ASIC3-like current activated at pH 6.0 by 1 mM and 3 mM tetracaine. (paired *t* test, *n* = 5; ***p < 0.001).

One potential limitation with the heterologous expression system is that it may not represent the effect of tetracaine on ASICs expressed in native neurons. To address this issue, we also investigated the potential effect of tetracaine on ASICs in mouse dorsal root ganglion (DRG) neurons. As reported previously [50,51], a biphasic ASIC3-like current was induced when the extracellular pH was lowered to 4.5 (Figure 6A), and addition of 1 mM tetracaine reduced the amplitude of the peak and sustained current activated at pH4.5 to 77.84% and 68.71% (Figure 6A-B). However, only the transient current component was visible when the extracellular pH was lowered to 6.0 as shown in Figure 6C. The amplitude of the peak current activated at pH6.0 was reduced to 74.23% by 1 mM tetracaine (Figure 6C-D). As expected, 3 mM tetracaine produced a greater reduction in the amplitude of ASIC currents. At pH4.5, for example, the peak and sustained currents were reduced to 68.71% and 48.24%, respectively (Figure 6C-D).

### Acid inhibits voltage-gated Na^+^ currents in primary cultured DRG neurons

**Figure 7:**
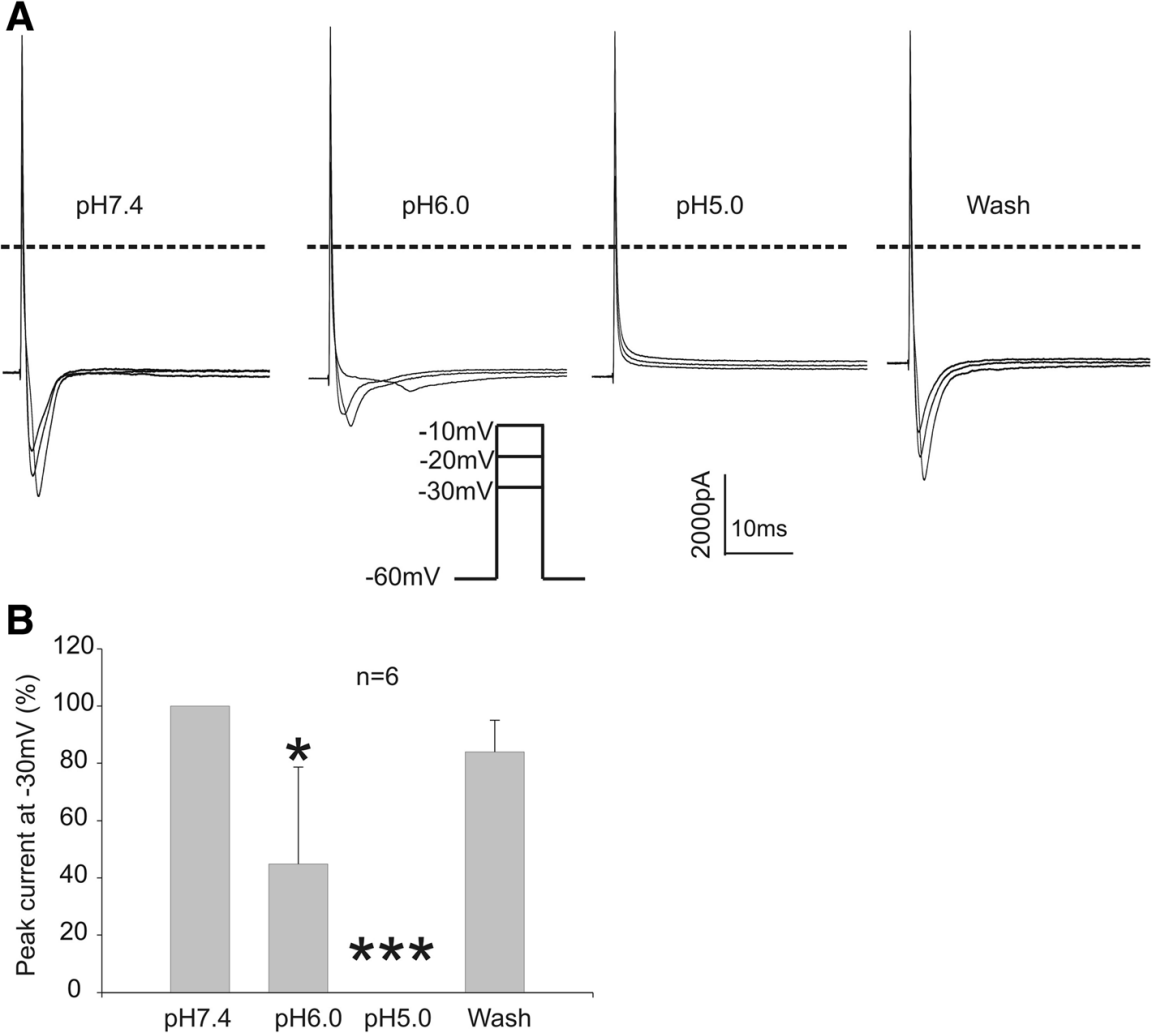
**Inhibition of voltage**-**gated Na**^+^**current by acid. A**. Representative current traces showing the inhibition of voltage-gated Na^+^ currents at pH 6.0 and 5.0 in DRG neurons. Neurons were held at -60 mV and stepped to potentials between −30 and −10 mV with 10 mV increments. **B**. Summary data showing that the peak Na^+^ currents at −30 mV were inhibited by acid (paired *t* test, *n* = 6; *p < 0.05, ***p < 0.001).

To confirm the inhibition of voltage-gated Na^+^ current by acidosis in DRG neurons in our recording condition, we compared voltage-gated Na^+^ currents recorded in normal solution (pH7.4) with that in acidic solution (pH6.0 and 5.0). Voltage-gated Na^+^ current was induced by step depolarization of the membrane potential from −60 mV to −30, -20 and −10 mV. We found that the amplitude of the inward Na^+^ currents was significantly suppressed at pH6.0, with a 55.15% inhibition observed at −30 mV (n = 6). Surprisingly, no inward Na^+^ current was recorded at pH5.0 (Figure 7A-B).

## Discussion

Acid sensing ion channels are proton-gated cation channels [2,5], which play important roles in physiological processes such as synaptic plasticity, learning and memory, and pathological conditions such as brain ischemia, epilepsy, and pain [17,20,22,52,53]. Activation of ASICs, such as ASIC3 and ASIC1a, was implicated in pain sensation [16-19]. In particular, the activation of ASIC3 has been implicated in chronic pain sensation [35]. In contrast to ASIC1a which only conducts a transient inward current, ASIC3 can conduct a biphasic current: a rapidly desensitizing peak current and a sustained non-desensitizing current that lasts as long as the extracellular pH remains acidic [5,32,54]. The fast current component is likely related to the onset of pain sensation while the sustained current, which persistently depolarizes neuronal membrane, may be implicated in longer lasting pain sensation. The inhibitory effect of tetracaine on both components of ASIC3 current may disclose a potential new mechanism for its analgesic effects.

Under painful conditions, tissue pH may drop dramatically to different values depending on the location and the severity of the pathological conditions [33,55,56]. ASIC3 is one of the most sensitive ASIC subunits, which can sense a decrease of pH to 7.0 [57,58]. The significant inhibition of tetracaine on the ASIC3 current, whether at a slight acidosis of pH 7.0 or severe acidosis of pH 4.5, suggests that it may have analgesic effects in multiple painful disorders with different degrees of acidosis. The finding that tetracaine is more effective in reducing the ASIC3 current at pH 7.0 than pH 4.5 or 6.0 likely suggests that uncharged forms of tetracaine are more effective in inhibiting the ASIC current. It also indicates that, at pathological conditions with minor pH drops, lower concentration of tetracaine is needed to suppress ASIC3-mediated nociceptive responses. There has been convincing in vivo studies in animal and human that ASICs mediate the pain perception induced by tissue acidosis [59,60]. Inflammation, a condition of local persistent acidosis, has also been found to increase ASIC expression, which is believed to account for hyperalgesia[61]. Local anesthetics exert their primary action by blocking the nerve conductance. The effect is mediated primarily by the blockage of sodium channel from inside of the cell membrane [47]. In severe acidic conditions (e.g. pH < 7.0), the penetration of local anesthetics into neuron is dramatically reduced by decrease of the non-ionized form. Accordingly, its effect on voltage-gated sodium channels is diminished in severe acidic conditions [47]. In contrast to its action on sodium channels, our studies showed that tetracaine still has significant effect on ASIC current at pH level as low as 4.5. When injected to the local tissue with acidosis, the effect of tetracaine would be compounded for its action on sodium ion channel, ASICs, and potentially others. Tetracaine inherently renders solution acidic, which effect might counteract its inhibition on ASICs. Considering this, we tested the effects of 3 mM tetracaine without pH adjustment on ASIC3, ASIC1a and ASIC2a. We found the slight pH drop of 0.01 caused by 3 mM tetracaine didn’t prominently decrease the inhibitory effect of tetracaine on ASICs (data not shown), comparing with those with pH adjustment.

ASIC1a, the most abundant subunit in the central nervous system, is also distributed in the peripheral nervous system. Similar to ASIC3, the activation of ASIC1a is implicated in pain sensation [4,16,18,19]. We showed that tetracaine inhibited both the ASIC3 and ASIC1a currents. In addition, we found that tetracaine inhibited ASIC1a current in a frequency-dependent manner: the higher frequency the channels were activated the greater the inhibition occurred. This frequency-dependent inhibition of the ASIC1a current should preferentially suppress high-frequency pathological activation of ASIC1a currents, for example, during epileptic seizure activities. Run-down is a characteristic of ASIC1a current [62,63], which is prominent in the first 10–15 minutes of the recording. However, after this period, ASIC1a current reaches a relatively steady state. To exclude the potential interference by run-down phenomenon, the effect of tetracaine on the ASIC1a current was tested 20 min after the formation of whole-cell configuration when stable currents were recorded.

ASIC2a is another subunit of ASICs present in both central and peripheral nervous systems. It can form homomeric ASIC2a channels, and heteromeric channels with ASIC1a [64]. In contrast to ASIC3 and ASIC1a, ASIC2a currents were not inhibited by tetracaine at 1–3 mM. Interestingly, high concentrations of tetracaine (10 or 30 mM) produced a potentiation of the ASIC2a current. Because of immediate deterioration of the tight seal after challenging the cells with tetracaine at 100 mM or higher, we were unable to perform the full dose–response relationship study on ASIC2a as well as ASIC3 currents. Unlike ASIC1a and ASIC3, the role of ASIC2a in pain sensation was poorly understood. In contrast to ASIC1a and ASIC3, ASIC2a is insensitive to the drop of extracellular pH with a threshold pH of ~5.0 and pH_0.5_ of ~4.4 [11]. Such severe acidosis may rarely happen even under pathological conditions. Thus, the clinical implication of the potentiation of ASIC2a current by higher concentrations of tetracaine remains to be determined. Since ASIC2a currents can be potentiated by high concentrations of zinc [65], whether tetracaine can interact with zinc binding sites on this subunit could be an interesting study in the future.

ASIC1β is a short form of ASIC1b. It shares a high sequence similarity with ASIC1a. The difference between ASIC1a and ASIC1β lies in the first 175 aa that includes short intracellular N-terminus, transmembrane domain I and a short extracellular segment. Future studies using chimeric ASIC1a/1β that contains different parts of the ASIC1β subunit may help in identifying the specific domain and/or amino acids involved in the effect of tetracaine.

Besides homomeric ASIC3, heteromeric ASIC1a/3 and ASIC1b/3 could also participate in acid-activated current in native sensory neurons, but their electrophysiological properties cannot be distinguish from the homomeric channels [51]. The ASIC3-like current in DRG neurons that were inhibited by tetracaine could be mediated by a combination of homomeric and heteromeric ASIC3 channels.

Injecting a local anesthetic into tissues has been used to block pain transmission for over a century. Although it is generally believed that blockade of voltage-gated Na^+^ channels and nociceptive impulses in the peripheral nerve fibers mediate the effect of local anesthetics, other mechanisms are likely to be involved in their interruption of nociceptive conduction in the spinal cord. For example, bupivacaine inhibits substance P release with an IC_50_ of approximately 1 mM [66]. In addition, it blocks capsaicin-induced Transient Receptor Potential Vanilloid receptor type 1 (TRPV1) current, which plays an important role in the development of hyperalgesia after injury [67]. Interestingly, TRPV1 current could be activated and sensitized by lidocaine with an EC_50_ of 12 mM [68]. More complex, quaternary lidocaine derivative QX-314 exerts biphasic effects on TRPV1 channels, inhibiting capsaicin-evoked TRPV1 currents at lower (micromolar) concentrations and activating TRPV1 channels at higher (millimolar) concentrations [69]. A recent study also demonstrated that lidocaine is a potent blocker for *I*_h_ with an IC_50_ of 72 μM, suggesting a potential new mechanism for systemic analgesic actions of lidocaine [70].

It has been shown by several studies that the activity of voltage-gated sodium channels, one of the primary targets for local anesthetics, are dramatically inhibited by acidic pH [40-42]. These findings are confirmed by our study in DRG neurons (Figure 7). The outer ring carboxylates of sodium channel can be protonated in an acidic environment, which causes a significant reduction of the single-channel conductance [71]. At the same time, the protonation of local anesthetics under acidic conditions could strongly decrease their potency for block of Na^+^ current [47,72]. Thus, other molecular mechanisms may be involved in the analgesic effects of local anesthetics, particularly in conditions of severe acidosis where the activities of Na^+^ channels are already suppressed by protons. Our studies suggest that ASICs might be an alternative target.

Our previous study found that lidocaine inhibits the ASIC1a current in mouse cortical neurons at 1 mM concentration. Since ASIC1a is implicated in neurological disorders such as brain ischemia while lidocaine can be used systemically and showed neuroprotection in some studies, inhibition of the ASIC1a current could be a potential and alternative mechanism for its neuroprotective effect. However, the concentration of lidocaine required to inhibit the ASIC1a current is unlikely to be tolerable for systemic use owing to the potential neuronal toxicity reported even at a much lower concentration range [73]. On the contrary, local anesthetics are more commonly used in the peripheral nervous system as analgesic agents. Thus, the potential effect of local anesthetics on peripheral ASICs may have more clinical relevance. Tetracaine could inhibit ASIC3 and ASIC1a currents with a threshold concentration of 0.3 mM, and inhibit approximately 30% of the ASIC current in DRG neurons at 1 mM. The formulations of 1%-5% for local anesthetics in topical use correspond to about ~40-200 mM. For example, a previous study showed that tetracaine and its analog N-butyl tetracaine at 100 μM use-dependently inhibited ~80% of Na^+^ current measured at 30-s interval by the pulse protocol [44]. However, 37 mM of N-butyl tetracaine (equivalent to 1.11% tetracaine-hyprochloric acid concentration) was used to elicited sciatic nerve block. Another example showed that tonicaine and lidocaine could inhibit 55% and 27.1% of the Na^+^ current at 100 μM. However, in vivo injection of tonicaine at 1% lidocaine equivalent concentration (42.67 mM) was used to elicit complete functional block for withdrawal response to pinch [74]. This difference of effective concentrations between the in vitro and in vivo model might be caused by the factors such as the permeability of the neural sheath, the absorption or diffusion of these compounds in the surrounding tissues. Additionally, other mechanisms might be involved, for instance our study showed that tetracaine inhibits ASICs at mM concentrations which are more closed to the concentrations used in the above two studies. Although the final concentration in the local tissue is difficult to measure, even a 100 time dilution could result in millimolar concentration.

## Conclusions

ASICs are implicated in pain sensation, and local anesthetics have analgesic effects. In the present study, we demonstrated that tetracaine can frequency-dependently inhibit the ASIC1a current and concentration-dependently inhibits the ASIC3 current. Inhibition of ASIC1a and ASIC3 activity by tetracaine discloses a potential novel mechanism for the analgesic effects of tetracaine, particularly in acidic conditions where its conventional target (voltage-gated Na^+^ channels) has already been suppressed by H^+^.

## Methods

### Culture of CHO cells and ASICs transfection

CHO cells were cultured in 35 mm dishes with F12K medium (Invitrogen, Carlsbad, CA, USA) containing 10% fetal bovine serum (Invitrogen, Carlsbad, CA, USA), 50 units/ml penicillin, and 50 μg/ml streptomycin. At ~50% confluence, cells were transfected with complimentary DNA for rat ASIC1a fused with a green fluorescence protein (GFP) at the c-terminal [75], or cotransfected with complimentary DNAs for rat ASIC1β, ASIC2a or ASIC3 with that for GFP, as described previously [76]. GFP-positive Cells were used for electrophysiological recordings 24-72 h after transient transfection.

### Primary culture of DRG neurons

Dorsal root ganglia were dissected from embryonic Swiss mice at 16 days of gestation, enzymatically dissociated with 0.25% trypsin for 10 min, and plated in poly-D-lysine coated dishes. Cells were initially cultured in DMEM containing 10% fetal bovine serum (FBS) and 10% horse serum and maintained at 37°C in a humidified 5% CO_2_ atmosphere incubator. After 24 h, culture medium was replaced with Neurobasal medium supplemented with B27 and glutamax. The cultures were fed twice a week and used for electrophysiological recordings 6 ~ 8 days after plating.

### Electrophysiology

ASICs currents were recorded with the whole-cell patch-clamp and fast perfusion techniques, as previously described [22]. GFP-positive CHO cells were selected for the recordings of ASIC currents. As for DRG neurons, only those cells that have typical long processes as well as voltage-gated Na^+^ current were used for ASICs recording. For fast-perfusion, a multibarrel perfusion system (SF-77B, Warner Instruments, Hamden, CT) was used. Patch pipettes were pulled from borosilicate glass. Pipettes had a resistance of 2–4 MΩ when filled with the intracellular solution. Whole-cell currents were recorded using Axopatch 200B amplifiers (Axon Instruments, Foster City, CA). All data were filtered at 2 kHz and digitized at 5 Hz using Digidata 1320 DAC units (Axon Instruments). The on-line acquisition was done using pCLAMP software (version 9.2, Axon Instruments). The recordings with an access resistance of less than 10 MΩ and a leak current less than 100 pA at −60 mV were included for data analysis [75]. Extracellular acidic solution was applied for 1 s or 4 s as indicated, with an interval of 1.5 or 2 min. The maximal inward current value was measured as the peak current. The sustained current for ASIC3 subunits was measured at the end of 4 s perfusion of the acidic solution. Since ASIC1a currents show significant rundown in the first 10–15 min after establishing the whole-cell configuration, in general, the effect of tetracaine (Sigma-aldrich, Inc, St. Louis, MO. USA) was tested ~20 min after the formation of whole-cell configuration and following the recording of at least three stable ASIC currents

### Solutions and chemicals

Extracellular solution contained (mM): 140 NaCl, 5.4 KCl, 20 HEPES, 10 Glucose, 2 CaCl_2_, 1 MgCl_2_, pH 7.4 - 4.5, adjusted with NaOH and HCl, 320–330 mOsm. Intracellular solution contained (mM): 140 CsF, 1 CaCl_2_, 10 HEPES, 11 EGTA, 2 TEA, 4 MgCl_2_, pH 7.3, adjusted with CsOH, 290–300 mOsm [77]. Tetracaine hydrochloride was dissolved in pH 6.0 or pH 4.5 extracellular solutions. A slight drop of pH was noticed, which was corrected with NaOH.

### Statistical analysis

All data were expressed as mean ± SD. GraphPad Prism 4 and Sigma Plot were used for statistical analysis. ANOVA followed by Bonferroni posttests or student’s *t* test were used to examine the statistical significance. The criterion for significance was set at *p* < 0.05. The dose–response curve was fitted with 3 parameter logistic nonlinear regression model: *y* = a/(1 + (x/x0)^b), where a is the relative maximal current, x0 is C_50_ and b is the Hill coefficient.

## Competing interests

The authors declare that they have no competing interests.

## Authors’ contributions

TL carried out the electrophysiological recording, data analysis and drafting the manuscript. ZX designed, supervised the project and revised the manuscript. JL and JEC participated in the design of experiments and revised the manuscript. All authors contributed to data interpretation, and approved the final manuscript.
